# Supplementary material for: Clinical Safety of Expanded Hemodialysis Compared with Hemodialysis Using High-Flux Dialyzer during a Three-Year Cohort
Source: J Clin Med. 2022 Apr 18;11(8):2261. doi: 10.3390/jcm11082261 (PMC9025951; doi:10.3390/jcm11082261)
Supplement: Supplementary file 1 [file jcm-11-02261-s001.zip › jcm-1667447-supplementary.pdf]

Supplementary Table S1. The differences of laboratory parameter values according to the membrane types and time

	Membrane (MCO versus HF)			Time (per month)			Interaction between membrane and time		
	Estimate (β)	SE	*P*-value	Estimate (β)	SE	*P*-value	Estimate (β)	SE	*P*-value
Hemoglobin	0.092	0.178	0.608	0.004	0.005	0.418	-0.007	0.006	0.223
Ferritin[a]	0.294	0.163	0.076	-0.005	0.004	0.163	-0.009	0.005	0.066
Albumin	-0.180	0.053	0.001	-0.001	0.001	0.682	-0.0003	0.002	0.855
Calcium	-0.002	0.144	0.988	-0.012	0.003	<0.001	-0.004	0.004	0.320
Phosphorus	-0.177	0.259	0.495	0.007	0.006	0.261	0.010	0.008	0.188
Intact PTH[a]	0.183	0.181	0.316	0.012	0.003	0.001	-0.001	0.004	0.778

P-values of linear mixed models were approximated by Satterthwaite's method. [a]Ferritin and intact PTH values were log-transformed. MCO, medium cut-off membrane; HF, high-flux membrane; SE, standard error; PTH, parathyroid hormone.

Supplementary Table S2. The differences of drug prescriptions according to the membrane types and time

	Membrane (MCO versus HF)			Time (per month)			Interaction between membrane and time		
	Estimate (β)	SE	*P*-value	Estimate (β)	SE	*P*-value	Estimate (β)	SE	*P*-value
Darbepoetin[a]	-0.102	0.122	0.408	-0.003	0.009	0.726	0.013	0.011	0.258
Phosphate binder[b]	-0.312	0.610	0.611	-0.051	0.063	0.423	0.123	0.077	0.115
Cinacalcet[b]	0.260	0.487	0.597	0.074	0.067	0.278	-0.010	0.078	0.895
Paricalcitol[b]	0.021	0.408	0.959	0.022	0.038	0.573	-0.012	0.046	0.799

P-values of linear mixed models were approximated by the Satterthwaite's method. [a]Darbepoetin doses were log-transformed. [b]The quantities of phosphate binder, cinacalcet, and paricalcitol were square root transformed. Phosphate binder included calcium carbonate, calcium acetate, and sevelamer. HF, high-flux membrane; MCO, medium cut-off membrane; SE, standard error.

Supplementary Table S3. The differences of inflammatory cytokines levels according to the membrane types and time

	Membrane (MCO versus HF)			Time (per a year)			Interaction between membrane and time		
	Estimate (*β*)	SE	*P*-value	Estimate (*β*)	SE	*P*-value	Estimate (*β*)	SE	*P*-value
IFN-γ[a]	-0.019	0.215	0.929	-0.262	0.066	<0.001	0.040	0.082	0.626
IL-1β[a]	-0.064	0.179	0.719	-0.369	0.098	0.001	0.107	0.123	0.392
IL-6[a]	0.154	0.215	0.477	-0.011	0.068	0.877	-0.039	0.085	0.646
TNF-*α*	-1.046	0.746	0.163	-0.097	0.560	0.864	0.213	0.699	0.763

P-values of linear mixed models were approximated by Satterthwaite's method. [a]The levels of IFN-γ, IL-1β, and IL-6 were log-transformed. MCO, medium cut-off membrane; HF, high-flux membrane; SE, standard error; IFN, interferon; IL, interleukin; TNF, tumor necrosis factor.

Supplementary Table S4. The annual concentration values of inflammatory cytokines

	Baseline	Year 1		Year 2		Year 3	
	Mean ± SD (pg/mL)	Mean ± SD (pg/mL)	*P*-value	Mean ± SD (pg/mL)	*P*-value	Mean ± SD (pg/mL)	*P*-value
IFN-γ[a]	1.27 ± 0.60	1.16 ± 0.85	1.000	1.15 ± 0.80	1.000	0.48 ± 0.49	<0.001
IL-1β[a]	-0.24 ± 0.51	-0.43 ± 0.62	0.782	-0.85 ± 0.56	<0.001	-1.12 ±1.03	<0.001
IL-6[a]	1.74 ± 0.84	1.62 ± 0.66	1.000	1.62 ± 0.55	1.000	1.62 ± 0.69	1.000
TNF-α	24.42 ± 2.08	25.28 ± 2.59	1.000	25.18 ± 2.45	1.000	24.59 ± 7.77	1.000

P-values were calculated by pairwise paired t-test with Bonferroni correction, which analyzes the difference from the baseline value. [a]The levels of IFN-γ, IL-1β, and IL-6 were log-transformed. SD, standard deviation; IFN, interferon; IL, interleukin; TNF, tumor necrosis factor.
